# Combinatorial scaffold morphologies for zonal articular cartilage engineering^☆^

**DOI:** 10.1016/j.actbio.2013.12.030

**Published:** 2014-05

**Authors:** J.A.M. Steele, S.D. McCullen, A. Callanan, H. Autefage, M.A. Accardi, D. Dini, M.M. Stevens

**Affiliations:** a Department of Materials, Imperial College London, London, UK; b Department of Bioengineering, Imperial College London, London, UK; c Institute of Biomedical Engineering, Imperial College London, London, UK; d Institute for Materials and Processes (IMP), School of Engineering, The University of Edinburgh, Edinburgh, UK; e Department of Mechanical Engineering, Imperial College London, London SW7 2AZ, UK

**Keywords:** Cartilage, Repair, Scaffolds, Tissue engineering, Regenerative medicine

## Abstract

Articular cartilage lesions are a particular challenge for regenerative medicine strategies as cartilage function stems from a complex depth-dependent organization. Tissue engineering scaffolds that vary in morphology and function offer a template for zone-specific cartilage extracellular matrix (ECM) production and mechanical properties. We fabricated multi-zone cartilage scaffolds by the electrostatic deposition of polymer microfibres onto particulate-templated scaffolds produced with 0.03 or 1.0 mm^3^ porogens. The scaffolds allowed ample space for chondrocyte ECM production within the bulk while also mimicking the structural organization and functional interface of cartilage’s superficial zone. Addition of aligned fibre membranes enhanced the mechanical and surface properties of particulate-templated scaffolds. Zonal analysis of scaffolds demonstrated region-specific variations in chondrocyte number, sulfated GAG-rich ECM, and chondrocytic gene expression. Specifically, smaller porogens (0.03 mm^3^) yielded significantly higher sGAG accumulation and aggrecan gene expression. Our results demonstrate that bilayered scaffolds mimic some key structural characteristics of native cartilage, support in vitro cartilage formation, and have superior features to homogeneous particulate-templated scaffolds. We propose that these scaffolds offer promise for regenerative medicine strategies to repair articular cartilage lesions.

## Graphical abstract

**Figure:**
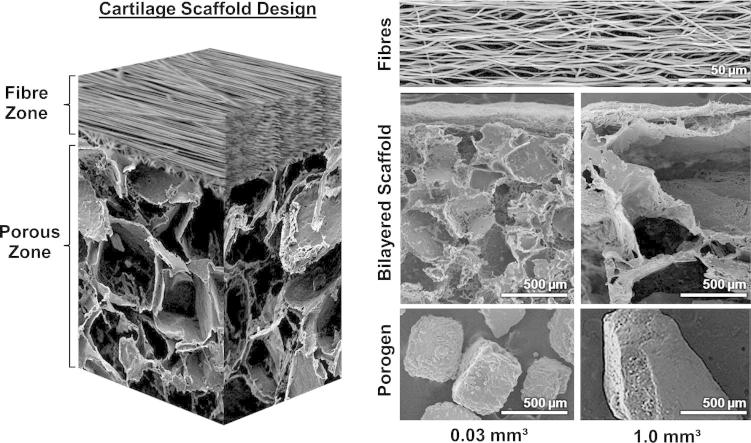

## 1 Introduction

Osteoarthritis is the predominant form of arthritis and remains a leading cause of disability [1]. Arthritic joints are characterized by lesions in hyaline cartilage that result in severe pain, loss of motion and eventually require surgical intervention [2]. Cartilage tissue engineering has emerged as a treatment method for articular cartilage lesions and this approach has employed a variety of scaffold materials which act as a carrier of and delivery vehicle for autologous chondrocytes and/or progenitor cells capable of cartilage formation [3–5]. Such strategies incorporate scaffold materials that range from autologous periosteal membranes to highly fibrillar materials derived from both biopolymers and degradable polymers including collagen, hyaluronic acid and aliphatic polyesters [6,7]. The materials used are predominantly isotropic in regards to functional characteristics including mechanical performance, cellular distribution, and cellular interaction with the matrix. Articular cartilage is a highly organized tissue that provides a low-friction and wear-resistant bearing surface and exhibits anisotropic mechanical properties as a result of depth-dependent differences in the density and structural arrangement of its extracellular matrix (ECM). Articular cartilage is comprised of four main zones: the superficial, middle, deep and calcified cartilage zone [8,9]. Each zone varies in regards to biochemical content, morphology and biomechanical function, with increased proteoglycan concentration and stiffness with depth, while conversely cellular density decreases; the total collagen concentration is unchanged with depth [8,10]. From a functional perspective, the cartilage portion of the osteochondral gradient can be simplified into two main regions: the superficial zone which exhibits a high tensile strength and low coefficient of friction to maintain smooth articulation; and a dense ECM region rich in proteoglycan molecules which contribute to the compressive mechanical properties by producing a high osmotic pressure within the tissue [5,11].

The superficial, middle and deep zones possess distinct gene and microRNA expression profiles, as thoroughly summarized by Grogan et al. [12]. When chondrocytes are expanded in vitro to obtain sufficient cell quantities for implantation, they dedifferentiate, losing their characteristic gene expression profile of collagen II and aggrecan by over an order of magnitude at the first passage, and begin expressing high levels of collagen I [13]. Dedifferentiation can be reversed by culturing chondrocytes on 3-D matrices, supplementation of the media with transforming growth factor (TGF)-β3 and dexamethasone, and physiological mechanical loading [14]. The redifferentiation protocols have been studied by gene expression as well as microRNA analysis, and in the case of microRNA expression can be almost entirely restored with the proper physical and chemical cues [13]. The use of zonally derived chondrocyte populations has merits in creating zonal constructs for implantation; however, the lack of efficient automated cell-sorting techniques limits their clinical applicability, leaving some to question whether zonal properties can be derived from an expanded redifferentiated chondrocyte population by simpler methods [15]. Beyond zonal populations, the use of purified progenitors isolated from cartilage using the classic stage-specific embryonic antigen-4 (SSEA-4) marker for undifferentiated stem cells has also proven of insufficient difference to warrant the additional complexity [16]. For this reason, a mixed population of passage-2 chondrocytes, cultured in a redifferentiation medium, remains the most widely investigated cell population for clinically applicable cartilage tissue engineering.

Current strategies for scaffold fabrication which have been shown to support cartilage formation include electrospinning of fibrous materials, particulate-leaching, gas-foaming and phase separation [9,17–21]. Each of these techniques offer specific advantages, yet none can fully encompass all requirements for optimal scaffold performance including zonal organization, adequate mechanical properties, full cellular ingress and physiological levels of ECM formation. Electrospinning is a facile technique which produces flexible, densely packed fibre networks with tuneable mechanical, physical and biological properties based on polymer selection, fibre size, network orientation and overall thickness [22,23]. Previous research has shown that aligned electrospun fibres are able to mimic the highly oriented morphological and tensile properties of the superficial zone of articular cartilage, and have been shown to support in vitro cartilage formation and chondrogenesis [18]. Nevertheless, electrospun scaffolds are generally limited in regards to dimensional thickness and lack of cellular infiltration due to the dense packing of fibres [22,24]. One exception is melt electrospinning, which can completely eliminate these issues, but concedes fine (<10 μm) fibre sizes for control, forming scaffolds intermediate between solution electrospinning and fused deposition modelling [25]. Particulate-templated scaffolds offer their own unique advantages including greater control of pore size, interconnectivity, geometry and overall scaffold porosity based on the size, shape and loading of the particulate [20].

Electrospinning directly onto a foam substrate produces a bilayered scaffold that possesses the tensile strength and fibrous morphology of the electrospun membrane and the high porosity of the underlying foam. The two-zone approach has been previously applied to vascular and bladder scaffolds which utilize the layered design to mimic the structural variations within their respective tissues [26–28]. As previously stated, articular cartilage also possesses a depth-dependent multi-zone morphology, with varied mechanical properties and function. A scaffold that mimics the structure of the native cartilage may offer a superior template for in vitro cartilage formation and ECM organization. Some approaches to multi-layer articular cartilage scaffolds include multi-layer hydrogels [29], multilayer electrospun fibres [18] and anisotropic fused deposition modelling [9]. This investigation is the first to study a zonally organized articular cartilage scaffold with an electrospun fibre articulating surface deposited onto a particulate-leached foam.

Herein, we generate bilayered poly(ε-caprolactone) (PCL) scaffolds comprised of an aligned fibre zone, which mimics the morphology and mechanics of the superficial zone of articular cartilage, laminated to a bulk porous particulate-templated scaffold which allows for full cellular infiltration and extensive ECM deposition. We hypothesized that the addition of aligned electrospun fibres would increase tensile properties, reduce surface roughness, and provide morphological similarities to native articular cartilage. Additionally, we varied the particulate size (0.03 vs. 1 mm^3^) in the particulate-templated scaffolds and hypothesized that smaller pores would support in vitro cartilage formation by bovine chondrocytes. Our results demonstrate that the electrostatic deposition of fibres with aligned orientation onto particulate-templated foams produces scaffolds which mimic key mechanical and functional characteristics of native cartilage and are able to support in vitro cartilage formation, indicating their promise in regenerative medicine strategies to repair articular cartilage lesions.

## 2 Materials and methods

### 2.1 Scaffold fabrication

#### 2.1.1 Particulate-templated scaffolds

PCL with a number average molecular weight, M_n_, of 80,000 Da, 1,1,1,3,3,3-hexafluoroisopropanol (HFIP) and dichloromethane (DCM) were procured from Sigma–Aldrich (Dorset, UK). Particulate-templated scaffolds were fabricated by mixing 30 g NaCl of varying sizes (0.03 or 1 mm^3^) with 8% w/v PCL in DCM to form a homogeneous paste that was cast into a custom aluminium mould (2 mm × 140 mm × 240 mm). The solvent was allowed to evaporate overnight, salt was extracted for 3 days in distilled water with water changes every 12 h, and scaffolds were air-dried for 1 day. Macroscopic sheets were formed with a final thickness of ∼2 mm.

#### 2.1.2 Bilayered fibre zone (FZ)/particulate-templated zone (PZ) scaffold construction

Particulate-templated scaffolds (PZ) were mounted onto a voltage-driven rotating mandrel (diameter 6.4 cm, length 10 cm) and aligned fibres (FZ) were deposited by electrospinning 5 ml of 12% w/v PCL in HFIP using a custom-made electrospinning setup that included a programmable syringe pump (Kd Scientific Model KDS 100 CE, Sandbach, Cheshire, UK) operating at 2 ml h^−1^, a Glassman high-voltage power supply series WR (Glassman, Bramley, Hampshire, UK) operating at 16 kV with an air gap distance of 11 cm, and a variable speed mandrel rotating at a linear velocity of 10 m s^−1^. Bilayered fibre scaffolds had a fibre membrane thickness of ∼200 μm with a fibre diameter of 1 μm.

### 2.2 Scaffold characterization using electron microscopy

Scaffolds were imaged by scanning electron microscopy (SEM) using a JEOL 5610 (Herts, UK). Specimens were coated with 100 Å Au using an Emitech K550 sputter coater and imaged at an accelerating voltage of 15 kV and a working distance of 15 cm. Cross-sectional examination of scaffolds was performed on freeze-fractured scaffolds prepared in liquid nitrogen.

### 2.3 Mechanical characterization of scaffolds

Particulate-templated and bilayered scaffolds were mechanically tested in tension to failure using an Instron Model 5540 testing machine (Norwood, MA, USA), equipped with a 50 N load cell operated at a crosshead speed of 10 mm min^−1^. Specimens had a gauge length of 30 mm and width of 10 mm; thickness was measured by digital callipers. Ultimate tensile strength and Young’s modulus were determined from stress–strain curves where the ultimate tensile strength was taken as the maximum stress and the Young’s modulus was calculated from the linear region of the stress–strain curve. All cellular and acellular mechanical measurements were performed with *n* ≥ 5 independent replicates.

Shear strength at the interface between the fibre and particulate-templated zones of the bilayered scaffolds was measured by carefully separating the individual zones of 50 mm length specimens to expose 10 mm of the particulate-templated zone or the fibre zone for fixation at either end, with a gauge length of 30 mm in between. Specimens were tested in both aligned and transverse fibre orientations; peak load (N) and stiffness (N mm^−1^) were determined.

Compression mechanics of bilayered scaffolds was assessed from 10 mm diameter samples that were pre-loaded to 0.05 N and compressed to 10% strain at a crosshead speed of 0.5% strain min^−1^. Following compression, samples underwent stress relaxation, which was quantified as the percentage decrease in force after 300 s at 10% strain. Tangent modulus and peak stress were calculated from the linear portion of the stress–strain curve and the maximum stress level achieved, respectively.

### 2.4 Bovine chondrocyte isolation

Bovine cartilage was harvested from the lower leg joints of a young calf. Briefly, the ankle joint was cut open along the joint line and cartilage tissue was cut with a scalpel into thin sections parallel to the subchondral bone. Chondrocytes were isolated by digesting in Dulbecco’s modified Eagle’s medium (DMEM) + Glutamax (4.5 g l^−1^ glucose) with 0.2% w/v pronase, 10 mM HEPES and 50 μg ml^−1^ gentamycin (all reagents from Invitrogen, Paisley, UK) for 1 h at 37 °C with agitation. This digest was removed and replaced with DMEM + Glutamax (4.5 g l^−1^ glucose) supplemented with 10 mM HEPES, 50 μg ml^−1^ gentamycin, 5% v/v fetal bovine serum (FBS) and 0.04% w/v collagenase (Sigma) overnight at 37 °C with agitation. After digestion, isolated chondrocytes were filtered through a 70 μm pore size filter, centrifuged at 250 x g for 3 min, and plated in DMEM (4.5 g l^−1^ glucose) with 10% v/v FBS, 50 μg ml^−1^ ascorbic acid (Sigma) and 50 μg ml^−1^ gentamycin (expansion medium).

### 2.5 Assessment of chondrocyte/bilayered scaffold interaction and in vitro cartilage formation

Particulate-templated and bilayered scaffolds were assessed for cell-seeding efficiency, cell infiltration and for cellular proliferation. Scaffolds 2 mm thick × 10 mm diameter were punched from macroscopic sheets. Scaffolds were sterilized in 70% ethanol for 30 min followed by three washes with sterile phosphate-buffered saline (PBS) and then soaked in 0.01% w/v bovine serum albumin (BSA) (Sigma–Aldrich, UK) in PBS overnight to promote chondrocyte adhesion. Bilayered scaffolds and control scaffolds (particulate-templated with no fibre membrane) were seeded with 1 × 10^6^ chondrocytes. A 20 μl aliquot of expansion medium containing 2 × 10^5^ chondrocytes (passage 2) was placed on the fibre side of each bilayered scaffold and cells were allowed to adhere for 2 h. Constructs were then inverted and the particulate-templated side was seeded with an additional 8 × 10^5^ chondrocytes in 80 μl of expansion medium, which were allowed to adhere for an additional 2 h before returning the constructs to the upright position and adding 2 ml expansion medium. Seeded scaffolds were cultured for up to 7 days and assessed for seeding efficiency, proliferation and chondrocyte distribution. Chondrocyte-seeding efficiency was assessed by comparing the DNA content of the seeded fibre zones (FZ-0.03/FZ-1.0) and particulate-templated zones (PZ-0.03/PZ-1.0) to the DNA content of 2 × 10^5^ and 8 × 10^5^ chondrocytes, respectively, as determined using a Quant-iT™ PicoGreen® kit (Invitrogen, UK), following the manufacturer’s instructions.

In vitro cartilage formation was assessed by culturing bilayered scaffolds seeded with 1 × 10^6^ chondrocytes, following the same seeding protocol, for up to 4 weeks in chondrogenic differentiation medium consisting of DMEM (4.5 g l^−1^ glucose), supplemented with 50 μg ml^−1^ l-proline (Sigma), 50 μg ml^−1^ ascorbic acid (Sigma), 0.1 mM sodium pyruvate (Sigma), 10 ng ml^−1^ TGF-β3 (Lonza, Slough, UK) and 1% v/v ITS Premix (BD Biosciences, Oxford, UK) at 37 °C and 5% CO_2_. Medium was changed twice weekly. Bilayered scaffolds were assessed at 1, 2 and 4 weeks after seeding. All biochemical assays were performed with *n* ≥ 4 independent replicates.

### 2.6 Chondrocyte biochemical assessment

After 0, 1, 2 and 4 weeks of culture, chondrocyte-seeded bilayered scaffolds were carefully separated into fibre and particulate-templated zones, freeze dried, and digested individually in papain digest solution (2.5 units papain ml^−1^, 5 mM cysteine HCl, 5 mM EDTA, in PBS (all reagents from Sigma Aldrich, UK)) at 60 °C overnight. Digested samples were assayed for total DNA content using a Quant-iT™ PicoGreen® kit and for sulfated glycosaminoglycan (sGAG) using the Blyscan Kit (Biocolor, Carrickfergus, UK) as per the manufacturers’ instructions. All biochemical assays were performed with *n* ≥ 4 independent replicates.

### 2.7 Mechanical testing and surface characterization of chondrocyte-seeded bilayered scaffolds

Tensile and compressive mechanics were assessed for acellular and for chondrocyte-seeded bilayered scaffolds at time points of 1, 2 and 4 weeks as described in Section 2.3. Surface roughness of particulate-templated and bilayered fibre scaffolds was assessed with white light interferometry (Wyko NT9100, Veeco) with a 20 × objective and digital images were captured over a 312 μm × 234 μm area with a *z*-axis resolution of 50 μm.

### 2.8 Histological examination of chondrocyte-seeded scaffolds

After 4 weeks in culture, bilayered scaffolds were fixed in formalin, dehydrated in an ethanol series and embedded in polyester wax (VWR, UK) at 45 °C. Sections were cut at 10 μm, adhered to Silane-Prep slides (Sigma Aldrich, UK) and dewaxed in Histo-Clear II (Fisher Scientific, UK). Dewaxed sections were stained for deposited sulfated proteoglycans (sGAG) using alcian blue (pH 2.5), collagen using picrosirius red, and with haematoxylin and eosin (H&E) for cell nuclei and extraneous matrix, respectively. For crossed polarized imaging of picrosirius red birefringence, the PCL was removed from sections with xylene overnight followed by an additional 30 min in chloroform to prevent the birefringence of PCL from hindering collagen fibril visualization. Immunohistochemical staining (IHC) was performed for collagen I (Abcam ab34710), collagen II (Abcam ab300) and collagen X (Abcam 58632) with rabbit IgG and PBS negative controls. Samples were pretreated with hydrogen peroxide, an avidin and biotin blocking kit (Vector Labs, UK), and blocked with 5% v/v goat serum. Primary antibodies were incubated overnight at 1/200 in 5% v/v goat serum, followed by goat-anti-rabbit HRP at 1:100 for 1 h, stained with a 3,3’-diaminobenzidine (DAB) kit (Vector Labs, UK) for 10 min, and counter-stained with haematoxylin. All stained sections were mounted with Histomount (Fisher Scientific, UK), and viewed on an Olympus BX51 microscope equipped with an Olympus DP70 camera.

### 2.9 Gene expression analysis

After 0, 1, 2 and 4 weeks, total RNA was isolated from the bilayered scaffolds (scaffold zones were separated prior to isolation using sterile forceps) by using an RNeasy kit and cDNA was obtained from reverse transcription using the QuantiTect Reverse Transcription kit (all from Qiagen, UK). Real-time quantitative polymerase chain reaction (qPCR) was carried out on a Rotorgene Corbett PCR, using previously published primer sequences. All qPCR measurements were performed with *n* ≥ 4 independent replicates. qPCR were performed on β-2 microglobulin (*B2M*), aggrecan (*ACAN*, U76615), collagen I (*COL1A1,* NM-174520) and collagen II (*COL2A1*, X02420) using the QuantiTect SYBR Green PCR kit in accordance with the manufacturer’s recommendations (Qiagen, UK). Primers, summarized in Table 1, were validated and the efficiencies of the primers were found to be between 0.90 and 1.07. The gene expression levels were normalized to the expression of the housekeeping gene *B2M* and were expressed as fold changes relative to week 0 passaged chondrocyte control samples (2 h post-seeding). The relative mRNA levels of *ACAN*, *COL1A1* and *COL2A1* were calculated using the ΔΔCt method and the *COL2A1/COL1A1* ratio corresponds to the 2^−ΔCt^ of each gene.

### 2.10 Statistical analysis

All experimental groups had a sample size of at least *n *= 4 for biochemical and qPCR and *n* ≥ 5 for mechanical property analyses. Data are presented as average ± standard deviation. Statistical significance was determined by performing one-way ANOVA with a significance accepted at *p*-value < 0.05.

## 3 Results

### 3.1 Fabrication of bilayered scaffolds

**Fig. 1:**
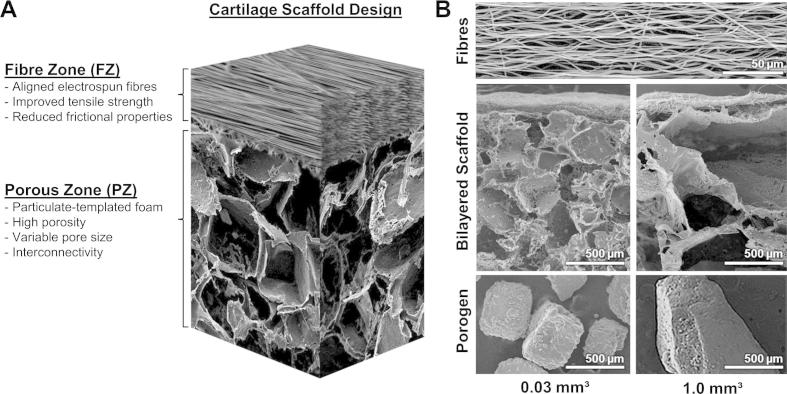
Bilayered cartilage scaffold schematic. (A) A diagram illustrating the electrospun fibre zone (FZ) deposited on a particulate-templated foam (PZ). The combination of the two distinct zones is designed to yield an anisotropic scaffold with a smooth articulating surface and a more porous region for ECM deposition. (B) Electron microscopy images of (top) the aligned fibre zone that is shared between both scaffold varieties, (middle) the complete bilayered scaffolds with 0.03 mm^3^ (left) and 1.0 mm^3^ (right) pores, and (bottom) the sodium chloride porogens used to produce their respective scaffolds.

Macroporous scaffolds were fabricated with two zones, the fibre zone (FZ) and porous zone (PZ) (Fig. 1A). The PZ was produced by particulate leaching of either 0.03 or 1.0 mm^3^ salt crystals embedded in a matrix of PCL followed by the electrostatic deposition of aligned PCL microfibres on their surface. Fig. 1B is composed of SEM micrographs of the aligned fibre layer, both bilayered scaffolds with fibre and porous zones (0.03 and 1.0 mm^3^), and the particulates used to create the differing pore architectures. Compressive properties of the particulate-templated scaffolds were influenced by particulate size with the smaller particulate (0.03 mm^3^) yielding a significantly stiffer scaffold (*p* < 0.005, Table 2), whilst both particulate-templated scaffolds had similar tensile properties (Table 2). Electrostatic deposition of aligned fibres on the particulate-templated scaffolds resulted in the adhesion of the fibre layer which significantly enhanced the tensile properties compared to the singular particulate-templated scaffolds (Table 2). Additionally, the surface roughness of the fibre layer was significantly lower compared to both particulate-templated scaffolds (Table 2). To assess the integration of the fibre layer with the particulate-templated scaffolds, interfacial mechanics were performed in both the aligned and transverse direction of fibre alignment. Interfacial stiffness was significantly higher in the aligned fibre direction (0.24 ± 0.04 N mm^−1^), relative to the transverse direction (0.08 ± 0.05 N mm^−1^) (*p *< 0.001). Peak loads of attachment for the 30 mm × 10 mm FZ–PZ interface were also significantly higher in the aligned direction than the transverse, with average values of 1.15 ± 0.3 and 0.47 ± 0.03 N respectively for BL-1.0 (*p* < 0.001) and 0.68 ± 0.03 and 0.430 ± 0.05 N respectively for BL-0.03 (*p *< 0.05).

### 3.2 Chondrocyte-bilayered scaffold interaction

**Fig. 2:**
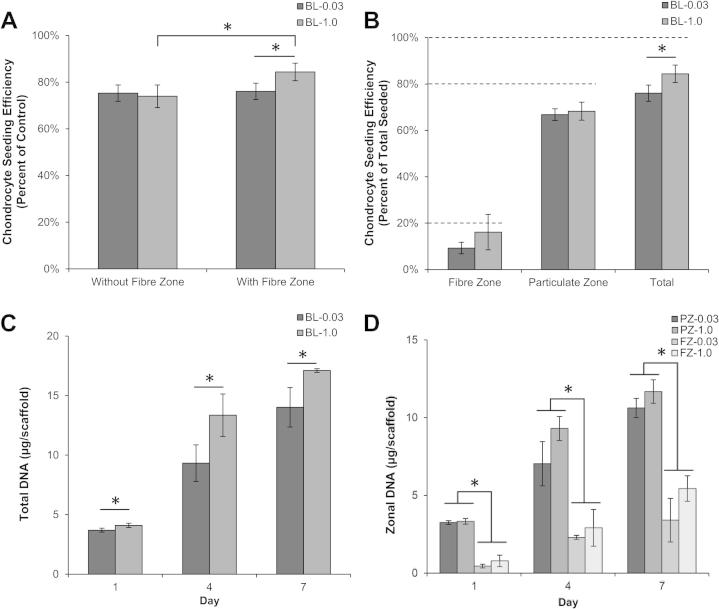
Chondrocyte-seeding efficiency and proliferation analyzed by DNA quantification. (A) Scaffold-seeding efficiencies with and without the fibre zone. Increased seeding efficiency was observed in BL-1.0 relative to BL-0.3 and both of the fibre-free scaffolds. (B) Seeding efficiency by scaffold zone showing the maintenance of the 20:80 FZ:PZ ratio at which they were initially seeded. (C,D) Total chondrocyte proliferation in the bilayered scaffolds (C) and subdivided into the FZ/PZ zones (D) over 7 days. ^∗^Significant difference (*p* < 0.05).

We first evaluated the ability of chondrocytes to adhere and proliferate on bilayered scaffolds for 7 days in vitro. Chondrocyte-seeding efficiency was assessed with and without the addition of the fibre zone to the particulate-leached scaffolds (Fig. 2). There was a significant increase in seeding efficiency from 74 ± 5% to 84 ± 4% in the BL-1.0 scaffolds with the addition of the fibre membrane (*p *= 0.045), but no significant difference in the BL-0.03 scaffolds, which averaged 76 ± 3%. Chondrocytes were well distributed throughout the scaffolds with and without the fibre membrane as assessed by DAPI staining (data not shown). Zonal seeding efficiency also demonstrated that chondrocytes were preferentially attached based on the seeding protocol with 9–16% of chondrocytes seeded on the fibre membrane, 66–68% seeded in the particulate-templated scaffold, yielding a total seeding efficiency range of 75–84%. Chondrocyte proliferation maintained this trend for both particulate scaffold types and demonstrated temporal increases in chondrocyte number as measured via DNA content (Fig. 2C, D).

**Fig. 3:**
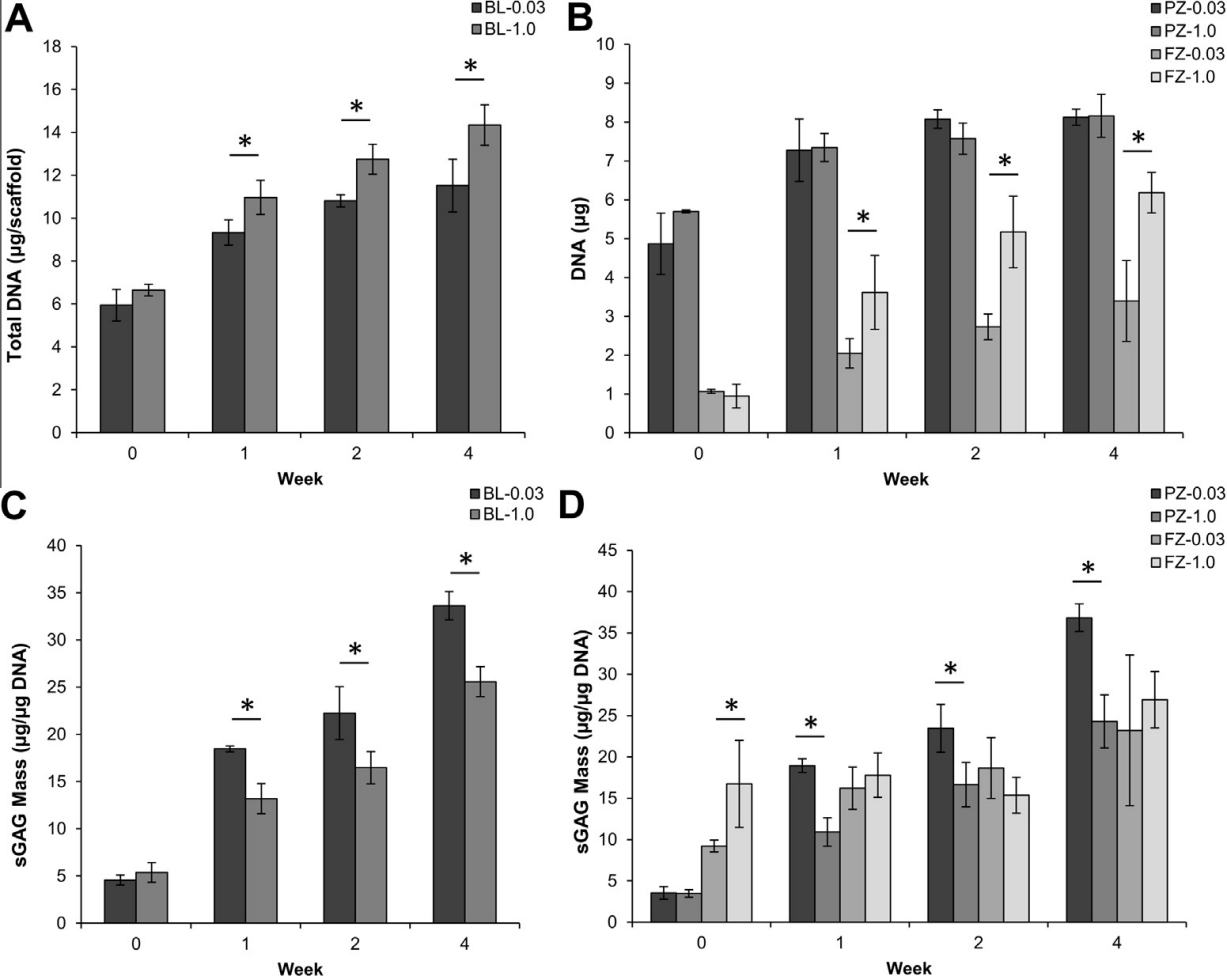
DNA and sGAG content in chondrocyte-seeded bilayered scaffolds over the 4 week culture period. (A) Total DNA content quantification per scaffold. (B) Zonal analysis of total DNA content. (C) Quantification of total sGAG per scaffold normalized to DNA content. (D) Zonal analysis of sGAG production normalized to DNA content. At all non-zero time points, the BL-1.0 had statistically higher proliferation due to differences observed in the fibre zone, while the BL-0.03 had statistically higher normalized sGAG production due to differences observed in the porous zone. ^∗^Significant difference (*p *< 0.05).

To evaluate in vitro cartilage formation, bilayered scaffolds were cultured for up to 4 weeks in chondrogenic medium*.* Chondrocytes were seeded with similar efficiencies on bilayered scaffolds of both porosities at week 0 and proliferated throughout the culture period. Total DNA was significantly higher for the bilayer-1.0 scaffolds vs. bilayer-0.03 at weeks 1, 2, and 4 (*p *< 0.05) (Fig. 3A). Zonal analysis demonstrated no significant differences between either PZ-0.03 or PZ-1.0, yet a significant increase in DNA content for the FZ-1.0 compared to FZ-0.03 at weeks 1, 2 and 4 (*p* < 0.5) (Fig. 3B). Sulfated GAG normalized to DNA content was also quantified as a marker of chondrocyte matrix accumulation, and increased in both a zonal and temporal fashion for both bilayered scaffolds with greater normalized accumulation in the BL-0.03 scaffolds at weeks 1, 2 and 4 (Fig. 3C). Total sGAG content was significantly higher for the bilayer-0.03 scaffold at weeks 1, 2 and 4 (*p *< 0.05). For the particulate-templated zones, there was significantly higher normalied sGAG accumulation on weeks 1, 2 and 4 for the PZ-0.03 yet no statistical differences for either fibre zone (Fig. 3D).

**Fig. 4:**
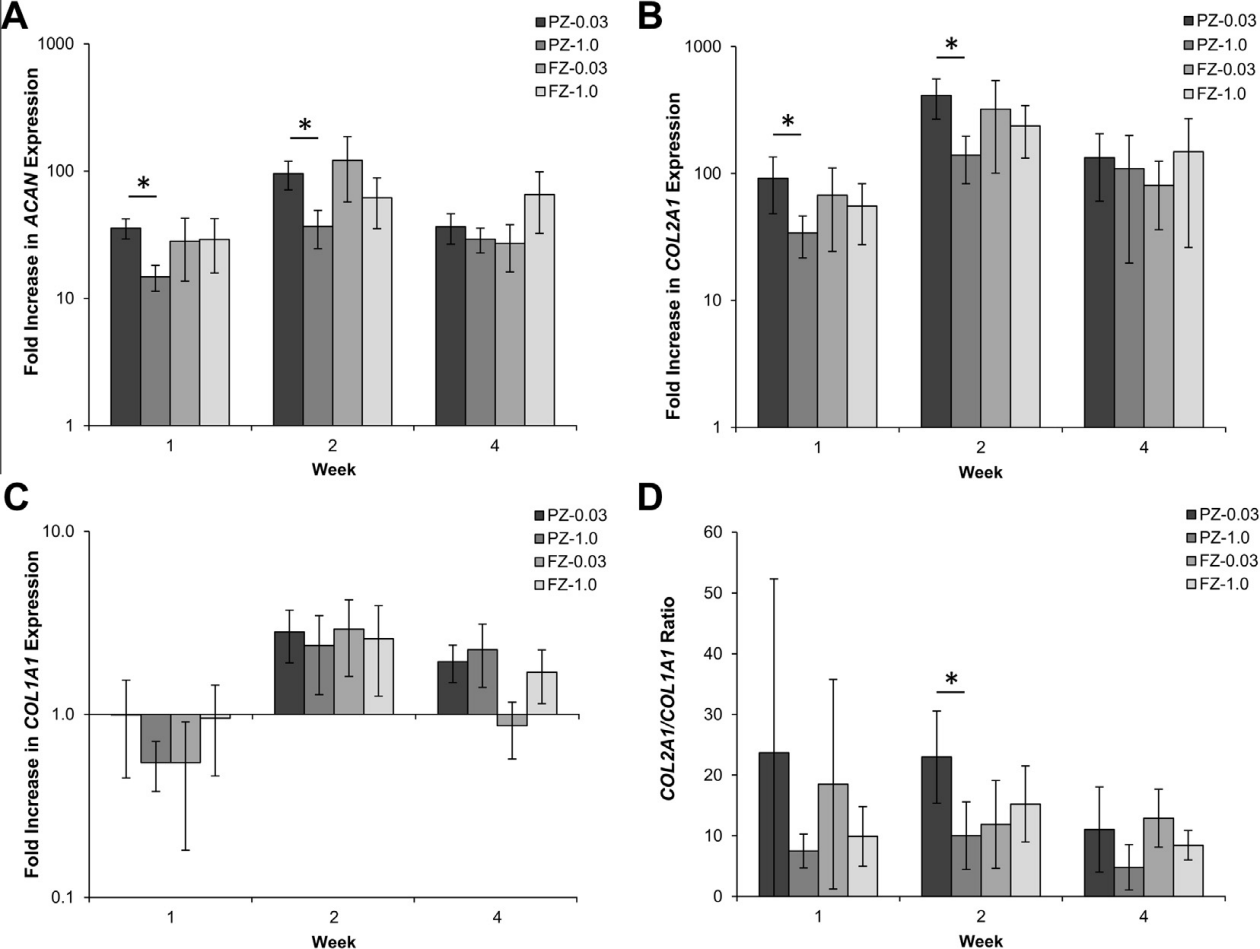
Zonal gene expression profiles of bovine chondrocytes cultured on the bilayered scaffolds in chondrogenic differentiation medium. mRNA levels of (A) *ACAN*, (B) *COL2A1* and (C) *COL1A1* are represented as fold difference relative to week 0 controls (2 h post-seeding) after normalization with the housekeeping *B2M* gene. (D) The *COL2A1*/*COL1A1* ratio was high throughout the culture period. ^∗^Significant difference (*p* < 0.05).

Gene expression profiles over the 4 weeks of culture in chondrogenic differentiation medium were quantified by scaffold zone (Fig. 4). mRNA levels of *ACAN* and *COL2A1* were significantly elevated at all time points compared to week 0 values and a significant peak in expression was observed at week 2 for both genes in the PZ-0.03 scaffold, while the other zones showed strong trends towards increased expression (Fig. 4A, B). The PZ-0.03 scaffold exhibited higher *ACAN* and *COL2A1* fold increase expression than the PZ-1.0 zone at both weeks 1 and 2 (*P *< 0.01 for *ACAN* and *P *< 0.05 for *COL2A1*). Expression of *COL1A1* was not significantly modified during the 4 week culture period and remained low (Fig. 4C). The differentiation index for chondrocytes (i.e. ratio of expression *COL2A1/COL1A1*) remained high throughout the culture period, with a significantly higher ratio for the PZ-0.03 at week 2 when compared to PZ-1.0, indicating maintenance of chondrogenic gene expression profile during the experimental time course (Fig. 4D).

**Fig. 5:**
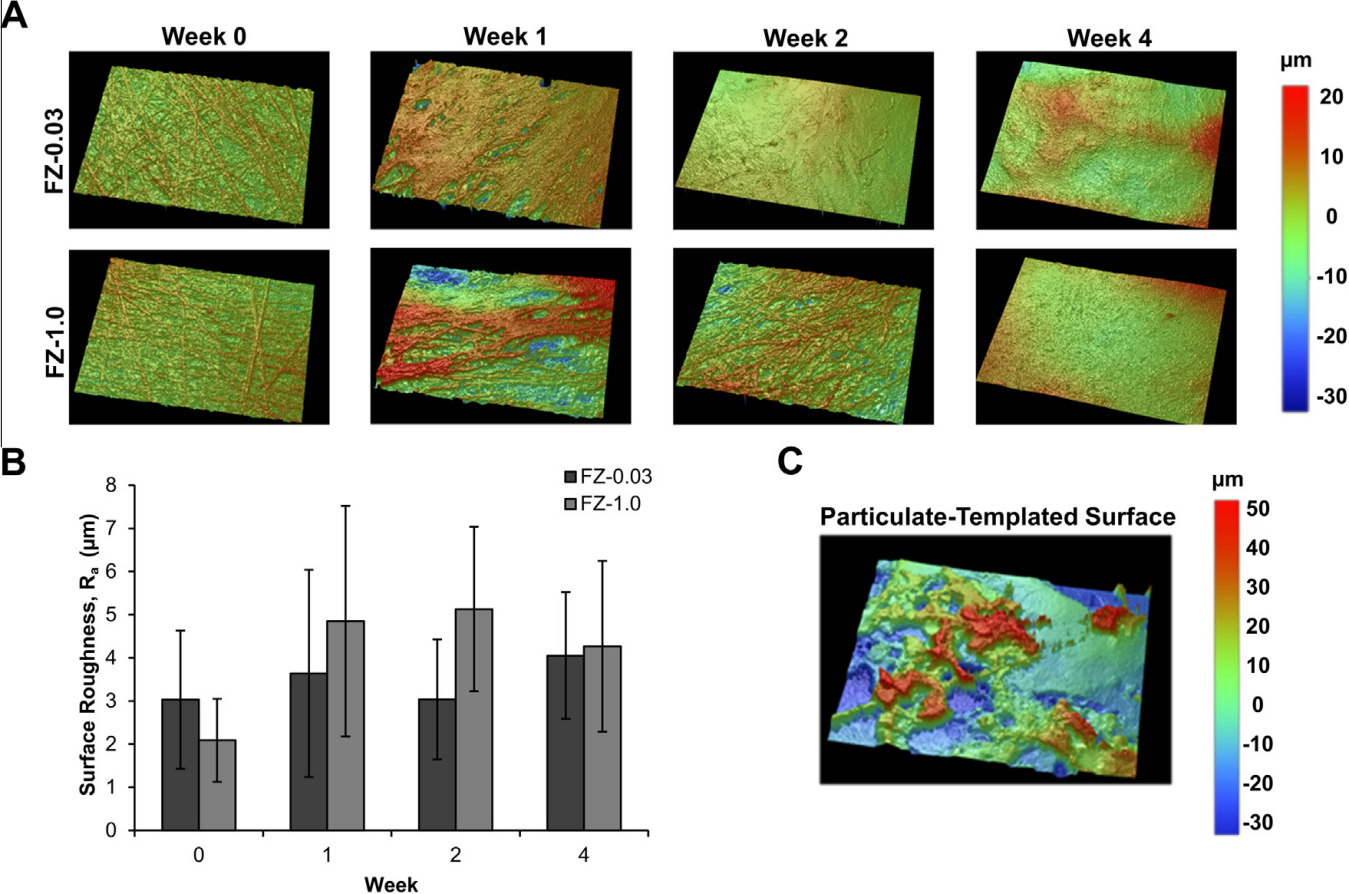
White light interferometry surface analysis. (A, B) Fibre zone surface roughness in bilayered scaffolds, analyzed over 4 weeks of culture. Representative areas measure 312 μm × 234 μm. (B) Surface roughness quantification of scaffolds with a fibre-zone. (C) Particulate-templated zone without a fibre-zone coating. The omission of the smooth fibre zone led to much greater surface roughness (R_a_) values ranging from 15 to 35 μm.

To evaluate temporal changes in surface roughness, white light interferometry was performed on the fibre zone and particulate-templated portions of the bilayered scaffolds on acellular samples and over 4 weeks of in vitro culture with chondrocytes (Fig. 5). The PZ-0.03 and PZ-1.0 mm scaffold surfaces were difficult to image as both exhibited high surface roughness with R_a_ values ∼15–35 μm (Table 2). The deposition of fibre zones onto both scaffolds resulted in significantly smoother surfaces compared to their respective particulate-templated counterparts with average R_a_ values of 3.0 ± 0.8 and 2.1 ± 0.4 μm for the FZ-0.03 and FZ-1.0, respectively. ECM accumulation was evident on the surface of the fibre zones throughout the 4 week culture period. Due to cellular infiltration and ECM deposition, the surface morphology was altered and the resulting R_a_ values slightly increased to 4.1 ± 0.7 and 4.3 ± 0.9 μm for the FZ-0.03 and FZ-1.0, respectively.

**Fig. 6:**
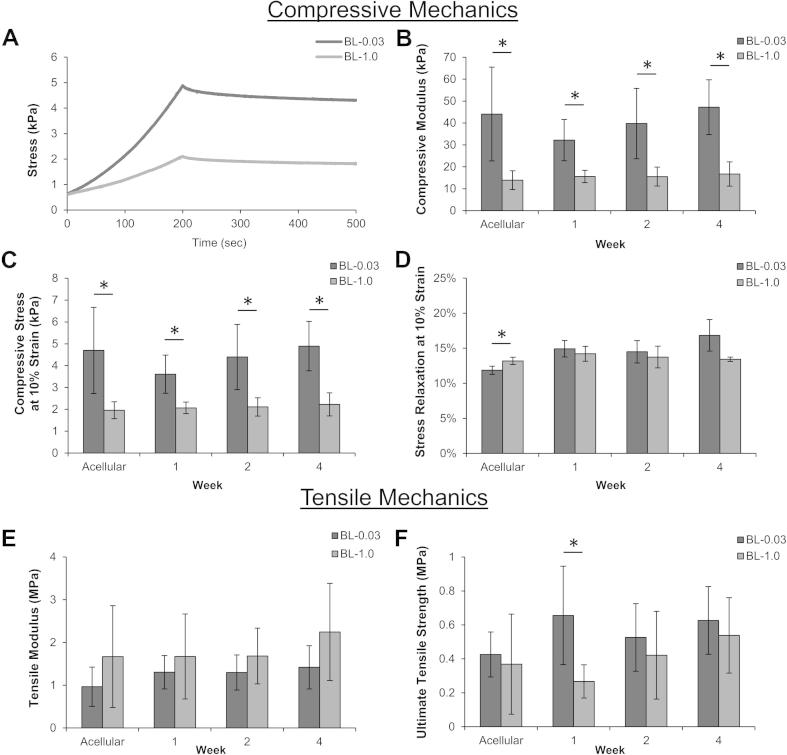
Compressive and tensile mechanics of bilayered scaffolds over 4 weeks of culture with chondrocytes. (A) Representative acellular responses for each scaffold under compression loading to 10% strain, followed by relaxation. Compressive modulus (B), stress at 10% strain (C) and stress relaxation (D) determined from the stress-relaxation test. Tensile modulus (E) and ultimate tensile strength (F) were not significantly different between the two scaffold groups. ^∗^Significant difference (*p *< 0.05).

Bilayered scaffolds were assessed for both compressive and tensile mechanics (Fig. 6). Compressive mechanics did not vary during the 4 week culture period and were similar to acellular controls, and the bilayer-0.03 scaffold was consistently significantly stiffer when compared to bilayer-1.0. Representative stress-relaxation curves demonstrated that the scaffolds were significantly different in regards to compressive modulus and compressive stress, yet both displayed similar relaxation profiles. Tensile mechanics of chondrocyte-seeded bilayered scaffolds displayed maintenance of tensile modulus and ultimate tensile strength throughout the culture period with no significant changes based on ECM deposition.

**Fig. 7:**
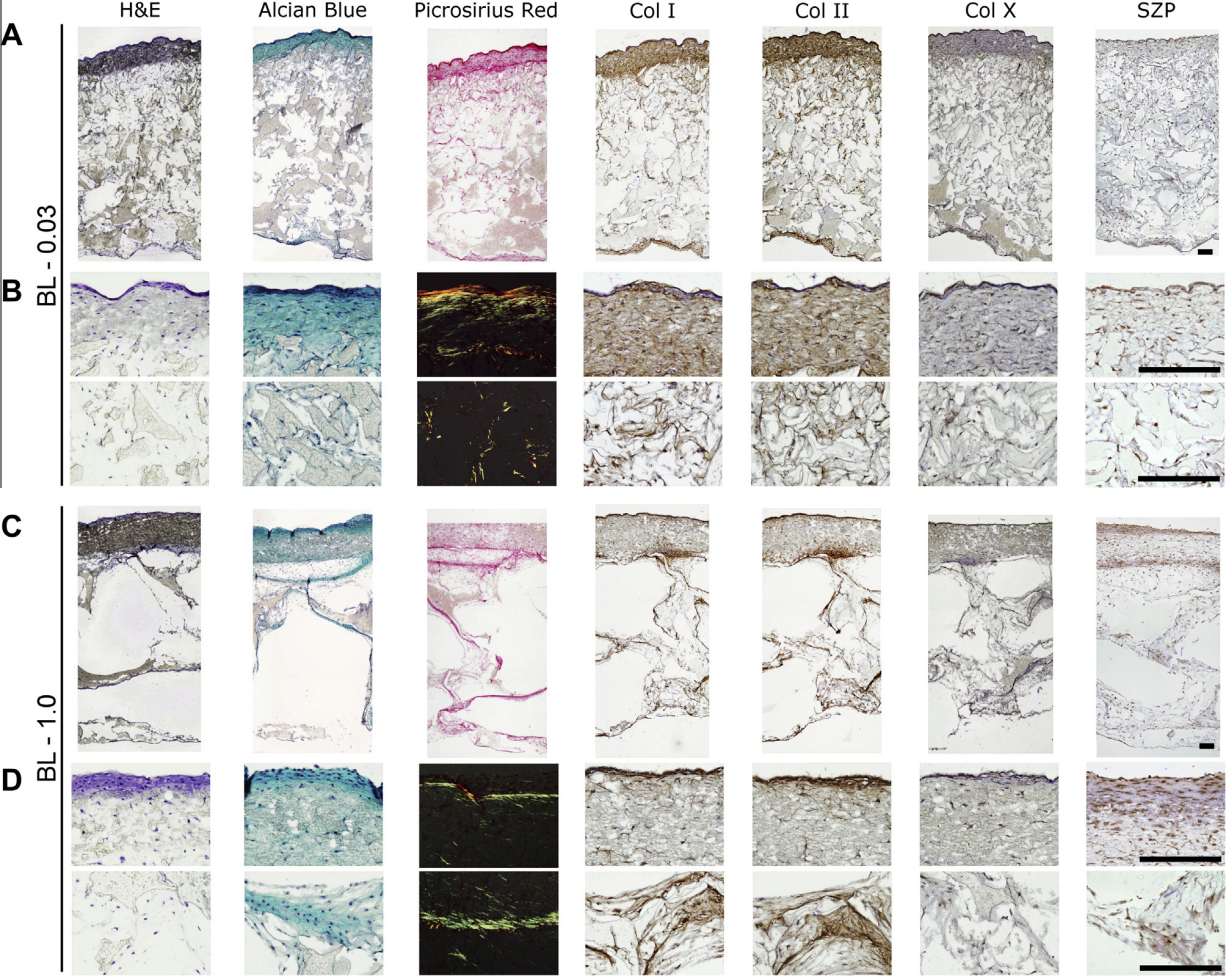
Histological examination of bilayered scaffolds BL-0.03 (A, B) and BL-1.0 (C, D) after 4 weeks in vitro*.* All scale bars represent 250 μm. Samples are shown at full thickness (A, C) and high-magnification FZ and PZ zones (B, D). Scaffolds are stained with haematoxylin and eosin, alcian blue for sGAG, picrosirius red for collagens, and by IHC for collagen I, II and X, and superficial zone protein (SZP), respectively, from left to right. The high-magnification images of the picrosirius red staining use cross-polarized microscopy, on sections where the PCL has been removed, to visualize collagen fibril birefringence.

Histological evaluation of the scaffolds after 4 weeks of in vitro culture indicated a dense cellular infiltration and ECM production in the fibre zone, intermediate cellular density and ECM production in the PZ-0.03, and low cellular density and ECM production in the PZ-1.0 (Fig. 7). Immunohistochemical staining showed a correlation between collagen I, collagen II and picrosirius red intensity, as would be expected from expanded P2 chondrocytes. Collagen X staining, an indicator of chondrocyte hypertrophy [30], is minimal. Superficial zone protein (SZP), a marker for the superficial zone [8], is found throughout the scaffolds within the pericellular matrix.

## 4 Discussion

Zonal organization of scaffolds that mimic the in vivo architecture and the structural design of cartilage are of crucial importance in regenerating the morphological as well as functional aspects of this challenging tissue. In this study, we have successfully fabricated bilayered cartilage scaffolds from PCL that possess zonal organization by utilizing a combinatorial strategy of electrostatic deposition of fibres on a particulate-templated scaffold. We selected PCL as it is used frequently in the field of musculoskeletal tissue engineering due to its biodegradable nature, facile processing capacity, elasticity and current use in FDA-approved medical devices [31]. To assess the performance of our scaffolds we investigated the in vitro cartilage formation of bovine chondrocytes. We performed zonal analysis of bovine chondrocyte attachment, proliferation and matrix production over 4 weeks in vitro as well as assessing the effect of particulate size (0.03 vs. 1.0 mm^3^) on chondrocyte gene expression, matrix accumulation and global scaffold mechanics. Our results demonstrate that the addition of aligned microfibres did not alter chondrocyte-seeding efficiencies (Fig. 2) and resulted in zonal differences in chondrocyte density and ECM formation (Fig. 3). Chondrocytes proliferated significantly on the FZ-1.0 zone, based on the large pores in the particulate-leached zone allowing complete and rapid cellular ingress and attachment to the fibre membrane. Despite the significant increase in chondrocyte number on the FZ-1.0 zone, sGAG content was not significantly different from the FZ-0.03 zone when normalized to DNA content (Fig. 3), signifying the importance of pore size on ECM production.

The addition of aligned microfibres significantly reduced the surface roughness of particulate-templated scaffolds and enhanced the tensile mechanics, regardless of particulate size (Table 2 and Fig. 5). Surface roughness (a key characteristic of the articulating surface for hyaline cartilage [32]) was evaluated using white light interferometry: the measurements of the topographical changes based on temporal evolution in ECM deposition showed that the scaffolds maintain a relatively smooth and increasingly homogeneous surface (maximum R_a_ = 4 μm). Particulate-templated scaffolds demonstrated high heterogeneity in surface roughness based on creation of large pores via particulate removal (Fig. 5). Tensile mechanics of the bilayered scaffolds were significantly enhanced based on the contribution of the tensile properties of the aligned fibres (Table 2). The enhanced porosity of the particulate-leached zone (PZ) and large pore size caused a significant decrease in the compressive modulus, when compared to the aligned electrospun membrane comprised of densely packed fibres. The benefit of our scaffold design is that both cellular ingress and ECM deposition in the PZ zone are possible, while the aligned fibre membrane provides a means to mimic the topographical, tensile, and frictional characteristics of articular cartilage. Moreover, it is our understanding that having the PZ zone with a slightly lower modulus should be favourable as this will not provide stress-shielding to cells as they experience compressive forces.

As indicated in Fig. 3B, we quantitatively determined that the chondrocytes were seeded in an 80:20 ratio at day 0. Over the next 4 weeks of culture, while there was proliferation within both zones, the DNA concentration within the superficial zone increased at a greater rate. As the fibre layer only comprises 10% of the total scaffold thickness, the initial seeding as well as the greater proliferation concentrated chondrocytes in the upper fibre layer, as is found in the native tissue, leading to increased ECM production in the superficial zone, as observed in the histological sections (Fig. 7).

Zonal analysis also revealed that a particulate size of 0.03 mm^3^ significantly upregulated expression of both *Aggrecan* and *Col2α1*, in PZ-0.03, and yielded the highest amount of proteoglycans as measured via sulfated GAG content. The benefit of the smaller pore size was localized to the particulate-leached zones, as the fibre zones from BL-1.0 and BL-0.03, which had identical morphologies, had similar gene expression profiles (Fig. 4). The observed increase in normalized sGAG production in the less porous PZ-0.03 is consistent with native tissue, wherein proteoglycan density has been correlated with decreased scaffold permeability and the resulting hypoxic gradients [33]. In both scaffold systems sGAG density was highest in the fibre zone, as observed by alcian blue staining intensity (Fig. 7). However, while the cellular density of the porous zone was lower than the fibre zone at 4 weeks in vitro, and proliferation had slowed (Fig. 3B), sGAG/DNA continued to increase (Fig. 3D). As the tissue had not yet reached a mature state, with time, the scaffold would be expected to fill with ECM and adopt a more deep-zone-like phenotype, characterized by low DNA and high sGAG concentrations [8,10]. The observed expression of SZP throughout the scaffold, in a non-zonal manner (Fig. 7), may be due to the use of a mixed population of chondrocytes cultured over 4 weeks in vitro in chondrogenic medium. Extended culture conditions have been shown to significantly increase expression of SZP in all zones of cartilage explants as well as in different chondrocyte morphologies including monolayer culture and inert hydrogel encapsulation [34]. Additionally, the lack of mechanical stimulation during culture would both have contributed to the lack of zonal SZP organization, leaving only the background chondrogenic-culture induced expression [35].

Lu et al. have shown that varying pore sizes in directionally frozen collagen scaffolds also affects cartilage formation—specifically, larger pores of 0.7 mm were ineffective in producing cartilage when seeded with bovine chondrocytes; however, in support of this work, pore sizes of 0.03 mm exhibited higher cell-seeding efficiencies and also more sulfated GAG per cell [36]. Tanaka et al. were able to vary both pore size and porosity of particulate-templated scaffolds using sugar crystals. Scaffolds of particulate size 0.03 mm^3^ and porosities of 90 or 95% yielded the best cartilage in terms of sulfated GAG content when compared to scaffolds formed from 1 mm^3^ size particulates, agreeing with our results presented here [37]. Mandal et al. demonstrated that layered scaffolds of varying pore size and morphology were able to support chondrocyte and fibroblast proliferation and matrix production, with smaller pores in the range of 60–80 μm producing more sGAG on a per cell basis after 4 weeks in culture [38]. A contrary observation has also been made by Woodfield et al. in melt electrospun scaffolds which show greater tissue production in large pores, attributed to nutrient availability, reduced cell–cell contact, and available voids to fill with ECM [9]. As tissue engineering investigations are highly multifactorial, the true cause of the differential ECM production is open to interpretation and warrants further investigation.

Our results demonstrate that morphological differences in bilayered scaffolds are able to contribute to gradations in chondrocytic gene expression and ECM deposition. By designing a scaffold organized into zones of differing mechanical and geometrical microenvironments, cellular-level changes have been shown to be imparted in a continuous construct. The addition of the aligned fibre membrane is able to enhance functionality of the salt-leached foam by reducing surface roughness and enhancing tensile mechanics while mimicking the orientation of the superficial zone of cartilage.

Bilayered scaffold structures have been used previously for blood vessel regeneration through the combination of different polymer systems. Here, we demonstrate that scaffold zones can be produced with different morphologies by utilizing the same polymer, and that these morphological changes are able to provide desired functionality while impacting both chondrocytic gene expression and ECM accumulation.

Previous studies have shown that a variety of scaffold morphologies can support in vitro cartilage formation including particulate-templated foams, electrospun fibrous scaffolds and rapid-prototyped scaffolds [9,17–21]. Electrospun fibres are able to act as functional membranes that can be engineered to have varying morphologies, mechanics and cellular responses, but standard solution electrospinning techniques are generally limited due to the fibres’ overall thickness as well as lack in porosity inhibiting full cellular colonization [18]. Particulate-templated scaffolds enable a high level of cellular ingress but can lack suitable mechanical properties based on their isotropic and highly porous nature. Combining these materials results in a zonally organized scaffold with benefits of both electrospun fibres (tensile/surface properties) and particulate-templated scaffolds (variation in pore size, porosity, pore interconnectivity and permeability). By taking a combinatorial approach to the use of different scaffold production techniques and morphologies we were able to yield engineered materials that provide zonal organization within a single scaffold system.

## 5 Conclusions

We fabricated bilayered scaffolds that mimic the functional organization of articular cartilage by the electrostatic deposition of fibres onto particulate-templated scaffolds. Our scaffolds overcome common limitations associated with homogeneous scaffolds by mimicking the functional interfaces of this tissue as well as supporting in vitro cartilage formation. Our analysis demonstrated zonal differences in cellular proliferation, biochemical composition and gene expression. Taken together, our results suggest that scaffolds inspired by the structural and functional organization of the native tissue may find use in developing regenerative medicine strategies to treat articular cartilage lesions.

## Figures and Tables

**Table 1:** qPCR primers.

Gene	Forward	Reverse
*B2M*	ACCTGCTGTCCCACGCTGAGT	TGTTCAAATCTCGATGGTGCTGCT
*ACAN*	GCTACCCTGACCCTTCATC	AAGCTTTCTGGGATGTCCAC
*COL1A1*	CATTAGGGGTCACAATGGTC	TGGAGTTCCATTTTCACCAG
*COL2A1*	CATCCCACCCTCTCACAGTT	GTCTCTGCCTTGACCCAAAG

**Table 2:** Properties of particulate-templated (PZ) and bilayered (BL) scaffolds produced with 0.03 or 1.0 mm^3^ porogens. Addition of the aligned fibre membrane in the BL scaffolds significantly enhanced tensile mechanics and lowered surface roughness when compared to single-component particulate-templated scaffolds.

Scaffold type	Tensile modulus (MPa)	Tensile stress at failure (MPa)	Compressive modulus (kPa)	Compressive stress (kPa) at 10% strain	Interfacial stiffness (aligned) (N mm^−1^)	Interface peak load (aligned) (N)	Surface roughness (R_a_; μm)
PZ – 0.03	0.04 ± 0.02	0.05 ± 0.01	40 ± 5	5 ± 1	N/A	N/A	16 ± 10
BL – 0.03	1.1 ± 0.3	0.4 ± 0.1	44 ± 21	4.7 ± 2.0	0.24 ± 0.04	0.68 ± 0.03	3.0 ± 0.8
PZ – 1.0	0.10 ± 0.05	0.03 ± 0.01	20 ± 5	2 ± 1	N/A	N/A	26 ± 20
BL – 1.0	1.6 ± 0.5	0.4 ± 0.2	14 ± 4	2.0 ± 0.4	0.23 ± 0.02	1.15 ± 0.03	2.1 ± 0.5
Fibre zone	28 ± 2	12 ± 3	140 ± 20	70 ± 4	N/A	N/A	3 ± 1

## Appendix A Figures with essential colour discrimination

Certain Figures in this article, particularly Figs. 5 and 7 are difficult to interpret in black and white. The full colour images can be found in the on-line version, at http://dx.doi.org/10.1016/j.actbio.2013.12.030
